# Phosphate Favors the Biosynthesis of CdS Quantum Dots in *Acidithiobacillus thiooxidans* ATCC 19703 by Improving Metal Uptake and Tolerance

**DOI:** 10.3389/fmicb.2018.00234

**Published:** 2018-02-20

**Authors:** Giovanni Ulloa, Carolina P. Quezada, Mabel Araneda, Blanca Escobar, Edwar Fuentes, Sergio A. Álvarez, Matías Castro, Nicolás Bruna, Rodrigo Espinoza-González, Denisse Bravo, José M. Pérez-Donoso

**Affiliations:** ^1^BioNanotechnology and Microbiology Lab, Center for Bioinformatics and Integrative Biology, Facultad de Ciencias Biológicas, Universidad Andres Bello, Santiago, Chile; ^2^Departamento de Bioquímica y Biología Molecular, Facultad de Ciencias Químicas y Farmacéuticas, Universidad de Chile, Santiago, Chile; ^3^Departamento de Ingeniería Química y Biotecnología, Facultad de Ciencias Físicas y Matemáticas, Universidad de Chile, Santiago, Chile; ^4^Departamento de Química Inorgánica y Analítica, Facultad de Ciencias Químicas y Farmacéuticas, Universidad de Chile, Santiago, Chile; ^5^Departamento de Ingeniería Química, Biotecnología y Materiales, Facultad de Ciencias Físicas y Matemáticas, Universidad de Chile, Santiago, Chile; ^6^Laboratorio de Microbiología Oral, Facultad de Odontología, Universidad de Chile, Santiago, Chile

**Keywords:** phosphate, quantum dots, nanoparticle biosynthesis, acid-stable quantum dots, bioleaching bacteria

## Abstract

Recently, we reported the production of Cadmium sulfide (CdS) fluorescent semiconductor nanoparticles (quantum dots, QDs) by acidophilic bacteria of the *Acidithiobacillus* genus. Here, we report that the addition of inorganic phosphate to *Acidithiobacillus thiooxidans* ATCC 19703 cultures favors the biosynthesis of CdS QDs at acidic conditions (pH 3.5). The effect of pH, phosphate and cadmium concentrations on QDs biosynthesis was studied by using Response Surface Methodology (RSM), a multivariate technique for analytical optimization scarcely used in microbiological studies to date. To address how phosphate affects intracellular biosynthesis of CdS QDs, the effect of inorganic phosphate on bacterial cadmium-uptake was evaluated. By measuring intracellular levels of cadmium we determined that phosphate influences the capacity of cells to incorporate this metal. A relation between cadmium tolerance and phosphate concentrations was also determined, suggesting that phosphate participates in the adaptation of bacteria to toxic levels of this metal. In addition, QDs-biosynthesis was also favored by the degradation of intracellular polyphosphates. Altogether, our results indicate that phosphate contributes to *A. thiooxidans* CdS QDs biosynthesis by influencing cadmium uptake and cadmium tolerance. These QDs may also be acting as a nucleation point for QDs formation at acidic pH. This is the first study reporting the effect of phosphates on QDs biosynthesis and describes a new cadmium-response pathway present in *A. thiooxidans* and most probably in other bacterial species.

## Introduction

Quantum dots (QDs) are fluorescent semiconductor nanoparticles made of bimetallic structures, showing unique luminescent and electronic properties (Valizadeh et al., 2012). The size and shape of QDs define their spectroscopic characteristics (absorption and emission spectra), and determine its application in different technologies such as fluorescent labeling and optoelectronics (Grieve et al., 2000; Monras et al., 2012; Subila et al., 2013). In particular, Cd-based QDs are used in electronic devices, in bioimaging applications (as a fluorophore conjugated to antibodies) and as photosensitizers in solar cells (Rühle et al., 2010; Valizadeh et al., 2012).

The use of microorganisms to biosynthesize QDs is of great interest because the nanoparticles produced in this way offer many advantages compared to chemical methods in terms of stability, water solubility, sustainable production and costs. The capacity to produce nanoparticles has been reported in distantly related bacteria such as *Escherichia coli, Bacillus mycoides*, and *Rhodopseudomonas capsulatus* (He et al., 2007; Iravani, 2014; Ordenes-Aenishanslins et al., 2014; Jacob et al., 2016). Biosynthesis of QDs has also been observed in bacterial cells with high metal resistance, increased tolerance to oxidative stress or overexpressing genes involved in thiol metabolism (Narayanan and Sakthivel, 2010; Valizadeh et al., 2012; Dunleavy et al., 2016; Ulloa et al., 2016).

Recently, we reported for the first time the production of CdS QDs in acidophilic cells (Ulloa et al., 2016). Quantum dots biosynthesis by extremophile bacteria belonging to the genus *Acidithiobacillus* was associated to Cd tolerance in a process favored by acidic pH (Ulloa et al., 2016). These QDs presented high stability at acidic pH and displayed diverse fluorescence properties, offering advantages to those produced by non-acidophilic bacteria or by chemical methods (Ulloa et al., 2016).

Acidophilic bacteria used in biomining processes have developed diverse strategies to cope with high metal ions concentrations, including active mechanisms also present in neutrophilic microorganisms, as well as passive mechanisms, such as complexation of metal ions with sulfate (Baillet et al., 1997; Dopson and Holmes, 2014). In addition, resistance to heavy metals like copper and cadmium has been related to the synthesis/degradation of inorganic phosphate granules (polyphosphate, or polyP) in *Acidithiobacillus ferrooxidans* (Dopson et al., 2003; Alvarez and Jerez, 2004; Martínez-Bussenius et al., 2017). In this model, when bacterial cells are grown in presence of high metal concentrations, there is a decrease in polyP levels and a concomitant formation of metal-phosphate complexes that contribute to detoxification (Dopson et al., 2003; Alvarez and Jerez, 2004).

As in *A. ferrooxidans*, polyP granules can be observed in other Acidithiobacilli, including *A. caldus* and *Acidithiobacillus thiooxidans* (Baillet et al., 1997; Orell et al., 2010; Navarro et al., 2013; Martínez-Bussenius et al., 2017), indicating the ubiquity of this polymer in these organisms. In addition, genes encoding the enzymes for polyP synthesis and degradation, PPK and PPX, involved in heavy metal resistance are also present in *A. thiooxidans* (accession numbers NZ_AFOH01000046.1 and NZ_AFOH01000004.1 for *A. thiooxidans* ATCC 19377 *ppx* and *ppk*, respectively) (Navarro et al., 2013; Martínez-Bussenius et al., 2017). Together, these data strongly suggest a conserved role for polyP in heavy metal resistance in this group of bacteria.

On the other hand, the production of QDs by *Acidothiobacillus* requires biological thiols like cysteine and glutathione for H_2_S generation (Ulloa et al., 2016). These antioxidant thiols have been previously described as precursors for Cd QDs in bacteria and fungus (Dameron et al., 1989; Bai et al., 2009; Valizadeh et al., 2012; Gallardo et al., 2014; Plaza et al., 2016). For example, in *Rhodopseudomonas palustris* the H_2_S produced by a cysteine desulfhydrase enzyme in presence of cysteine, allows the formation of Cd QDs (Bai et al., 2009).

The number of publications describing the biosynthesis of CdS QDs in bacteria has strongly increased during the last years (Plaza et al., 2016; Ulloa et al., 2016; Yan et al., 2017). However, the cellular mechanisms underlying the biosynthesis of CdS QDs are still unknown. In this context, a detailed study of the bacterial biosynthesis process might contribute to the understanding of the molecular mechanism underlying cadmium biomineralization. In particular, the study of these mechanisms in acidophilic bacteria used in biomining operations could contribute to the development of new bionanotechnological applications.

In this work, we used a Response Surface Methodology (RSM) to study the importance of different Cd and phosphate concentrations on *A. thiooxidans* ATCC 19703 QDs biosynthesis at acidic pH. RSM is a multivariate technique for analytical optimization that can be used to generate empirical models to optimize a response which is influenced by several independent variables. In addition, by using RSM we determined the effect of phosphate on cadmium uptake, tolerance, and polyP degradation in *A. thiooxidans* ATCC 19703. Based on these results, a mechanism explaining the role of phosphate on CdS QDs biosynthesis in *A. thiooxidans* ATCC 19703 is proposed.

## Methods

### Bacterial strain and growth conditions

All assays were performed with the acidophilic bacterial strain *A. thiooxidans* ATCC 19703 using growth conditions previously described (Ulloa et al., 2016). The culture medium used for bacterial growth was a basal medium adjusted to pH 2.3 [0.4 g/L (NH_4_)_2_SO_4_, 0.4 g/L MgSO_4_ × 7 H_2_O and 0.056 g/L KH_2_PO_4_], supplemented with spherical prills of elemental sulfur (S_8_) as an energy source (Espejo et al., 1988). The liquid cultures were grown for 7 days in flasks with constant agitation (150 rpm) at 28°C.

### Experimental design

In the present study, the experimental design and statistical analyses were performed according to Central Composite Design (CCD), a common and effective tool for determining relationships between independent variables and responses to build a second-order polynomial model. This model is then applied in RSM (Bezerra et al., 2008). Cadmium concentration (Cd^2+^) [X1] and phosphate concentration [X2] were chosen as independent factors. Cell number (determined by using a Petroff-hausser chamber, see protocol below) [Y1], Cd^2+^ uptake (intracellular Cd^2+^ determined by FAAS) [Y2], fluorescence [Y3], and polyP concentration (inorganic polyphosphate in microbial cells, see protocol below) [Y4], were selected as response variables. A quadratic polynomial model was used to identify all possible interactions of selected factors with response function as below:

$$\begin{array}{lll} \text{Y} & = & {\text{β}}_{0}+\beta_{1}^{*}\text{P}{\text{O}}_{4}+\beta_{2}^{*}\text{Cd}+\beta_{1.2}\left[\text{P}{\text{O}}_{4}\right]\left[\text{Cd}\right]+\beta_{1.1}{\left[\text{P}{\text{O}}_{4}\right]}^{2}\\ +\beta_{2.1}{\left[\text{Cd}\right]}^{2} \end{array}$$

where Y is the response, β_0_ is the constant coefficient, βi, βii, βij are the coefficients estimated by the regression for linear, quadratic and cross- product effects of X1, X2, and X3, respectively, on the response.

The CCD for two variables (cadmium and phosphate) was executed. This comprises experiments associated to three parts, *N* = 2^k^ + 2k + cp:

1. 2^k^: The factorial design part where k is the number of factors and 2 corresponds to the number of levels (−1 and +1).
2. 2k: The “star” or axial design part with levels −α and α. These experimental levels or points are equidistant from their center. The values depend on the number of variables and can be calculated by α = (2k) ^1/4^ For two variables the value is 1.41.
3. cp: Central point with level 0 for all variables.

Thus, all factors are studied in five levels (−α, −1, 0, +1, + α). This way, it is sought to increase the number of experimental levels for each factor and to describe in greater detail the response within the delimited ranges.

Two experimental regions were delimited and defined as follows:

- CCD for cadmium resistance: The *A. thiooxidans* ATCC 19703 cadmium minimum inhibitory concentration (MIC) previously determined by Ulloa et al. (2016) (200 mM), was considered as reference to select the cadmium concentrations tested (Ulloa et al., 2016). The range of phosphate concentrations was based on those used for CdS QDs biosynthesis (Table S1).
- CCD for QDs biosynthesis: The range of cadmium concentrations was defined according to the values in which QDs biosynthesis was observed, 10 mM. The range of phosphate concentrations was based on those used on CdS QDs biosynthesis (Table S1).

In both experimental regions, 9 conditions from the two studied variables were generated to model the response surface. This corresponds to 4 factorial points, 4 axial points, and 1 center point. Table S1 shows the nine coded conditions and the corresponding values of variables for the two experimental regions. Each experiment involves a total of 9 runs in triplicate. The RSM experiment was performed to examine the effects of cell number (biomass concentration), cadmium concentration, fluorescence emission (biosynthesis of QDs), and polyP degradation.

To generate a surface response from a quadratic model in the optimization of two variables, experimental values were analyzed using the statistical software (Statgraphics centurion XV). To test the statistical significance of the effects, ANOVA was the default analysis method used. The quality of the quadratic polynomial model fit was expressed by the coefficient of determination (*R*^2^), which is a key output of regression analyses. *R*^2^ is a measure of the amount of variation around the mean explained by the model. Statistical analyses such as Fisher' test (*F*-test), probability (*P*-value) at 95% confidence level, and alpha (5%) were used to assess the results.

### Quantum dots biosynthesis

Biosynthesis of CdS QDs was evaluated according to the protocol previously described by Ulloa et al. (2016). Briefly, cells were grown to stationary phase in basal media at pH 2.3, sedimented by centrifugation and then resuspended in phosphate buffer pH 3.5 to reach an OD _620 nm_ 1.3 (~1 × 10^11^ cells /mL), in the presence of cadmium (0.33–10 mM) and glutathione 5 mM for 24 h. Samples were then centrifuged 5 min at 10,000 × g and the fluorescence of supernatants was evaluated after excitation at 360 nm using a UV-transilluminator. Emission spectra of biosynthesized CdS QDs was measured using a Synergy H1M fluorimeter after excitation at 360 nm. QDs fluorescence on RSM studies was determined in supernatants.

### Quantification of Cd inside cells

Bacterial pellets obtained from cultures exposed 24 h to the conditions of interest were dissolved using 7 N HNO_3_ during 16 h at 37°C. 100 μL samples were mixed with 3.9 mL miliQ water and Cd was quantified in a flame emission spectrometer AA6200 (Shimadzu). Cd calibration curve was built using a commercial Cd standard (Sigma Aldrich). The intracellular Cd content was normalized by the number of cells.

### Quantification of cells

The number of cells was determined by using a Petroff -Hausser chamber model Thoma (double grid, 0.01 mm depth and 0.0025 mm^2^ surface area) as described before (Ulloa et al., 2016). The detection limit of this technique is 6.25 × 10^5^ bacteria/mL.

### Polyphosphate determination

Direct quantification of polyP was performed in cells exposed 24 h to biosynthesis conditions following the protocol described by Kulakova et al. (2011). Cells were adjusted to a concentration of 1 × 10^11^ cells/mL and then centrifuged 10 min at 8000 × g. Cell pellets were resuspended in 50 mM HEPES buffer (pH 7.4), kept at −80°C for 15 min and then heated at 60 °C for 10 min. Subsequently, cell pellets were resuspended in 20 mM HEPES/150 mM KCl assay buffer for subsequent addition of 2-(4-amidinophenyl)-1H-indole-6-carboxamidine (DAPI) (17 μM final concentration) and incubated 5 min at 37°C. Quantification of DAPI-polyP complex was performed by measuring the fluorescence at 550 nm (excitation at 415 nm). Since biosynthesized CdS QDs do not emit fluorescence when excited at wavelengths >400 nm (Monras et al., 2012), the fluorescence obtained is purely due to DAPI-polyP complex. Intracellular polyP levels were normalized by protein concentration. A commercial phosphate polymer (75 residues) in glass beads (Sigma Aldrich) was used as a control for the calibration curve and validation of the technique.

### TEM and EDS

Transmission electron microscopy (TEM) measurements were made using a FEI Tecnai G2 F20 S-Twin microscope, operated at 200 kV. For these studies, a drop of the dispersed sample was left to dry out on a commercial carbon coated Cu TEM grid. TEM images were processed and analyzed with Digital Micrograph 3.9.0 (Gatan Inc) and The Gimp 2.4.0 software packages. In addition, samples were chemically characterized by Energy-dispersive X-ray spectroscopy (EDS or EDX).

## Results

### Effect of phosphate on *A. thiooxidans* CdS QDs biosynthesis at acidic pH

In a previous work we determined that *A. thiooxidans* has the capacity to biosynthesize CdS QDs at different pHs (Ulloa et al., 2016). By means of DLS and TEM analysis we determined that QDs biosynthesized by acidophilic bacteria display a nanocrystal size below 20 nm, absorption peak at 360 nm and a broad emission spectra (450–650 nm) when excited at 370 nm (Ulloa et al., 2016). A uniform population of nanosized material was present in pellets and supernatants of *A. thiooxidans* cells exposed to biosynthesis conditions, with an average size of 6.9 and 10 nm for green and red nanoparticles, respectively (Ulloa et al., 2016). Here, we studied the effect of phosphate on the biosynthesis of cadmium nanoparticles, based on its importance on the interaction of acidophilic bacteria with metals (Dopson et al., 2003; Alvarez and Jerez, 2004).

**Figure 1:**
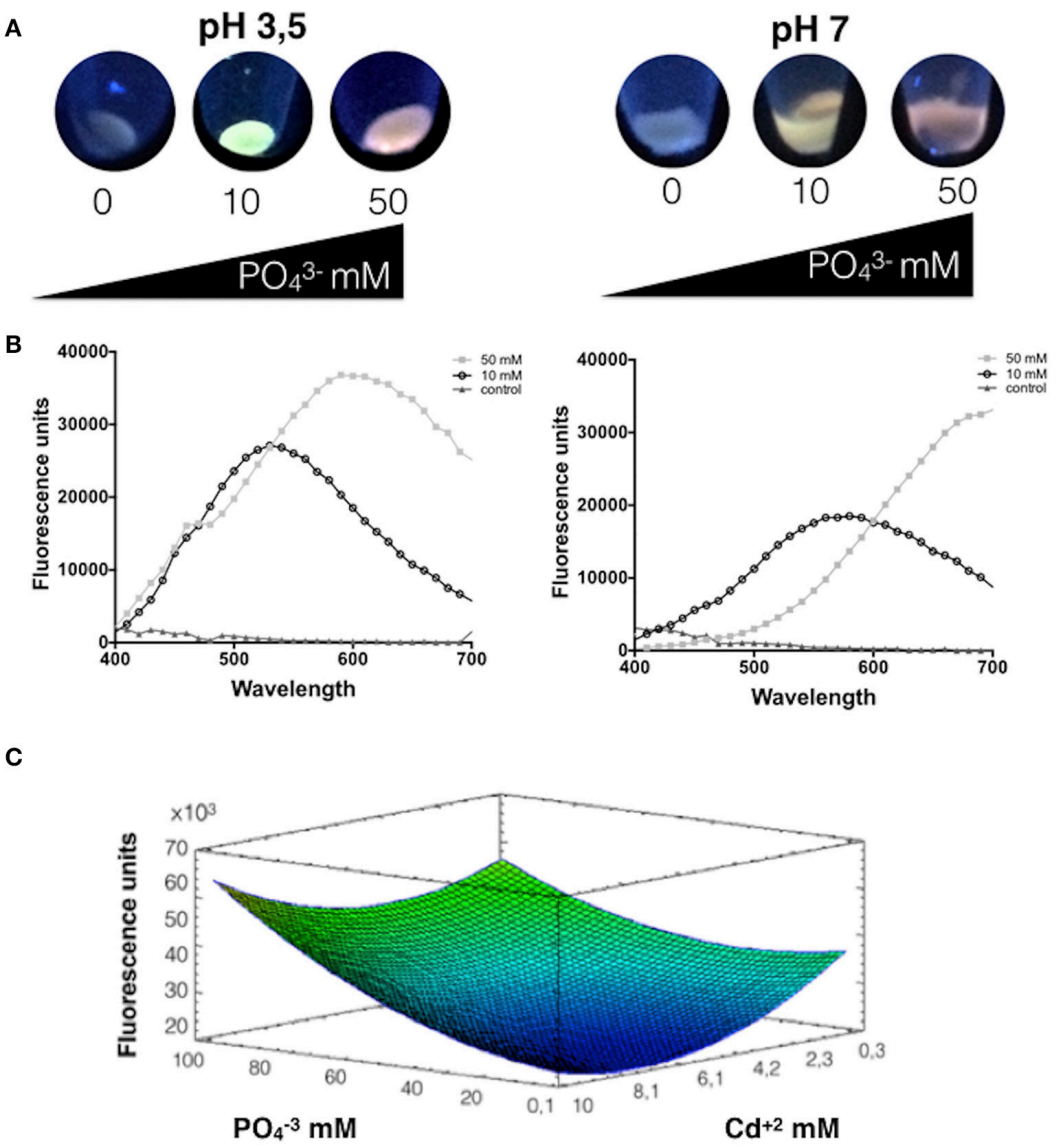
Phosphate favors the biosynthesis of CdS-QDs at pH 3.5. **(A)** Fluorescence of cells exposed to CdS-QDs biosynthesis conditions during 24 h in presence of different phosphate concentrations (Cd^2+^ 0.66 mM and phosphate 0, 10, or 50 mM). Fluorescence was evaluated after exposure to UV light (360 nm). **(B)** The emission spectra show the fluorescence peaks for each condition at pH 3.5 (left) and pH 7.0 (right). **(C)** Response surface for a two variables model for the effect of cadmium and phosphate on *A. ferrooxidans* QDs production in supernatants, determined as fluorescence emission (*R*^2^: 72%; *p* < 0.05). Warm colors represent an increase in value for the variable under study.

Phosphate showed to play a crucial role in CdS-QDs biosynthesis in *A. thiooxidans* ATCC 19703 at pH 3.5 and 7.0, since no fluorescence was observed in the absence of phosphate (Figure 1A). Fluorescent nanoparticles were observed in cell pellets after 24 h at pH 3.5 or 48 h at pH 7.0. Interestingly, variable emission colors and intensities were determined at different phosphate concentrations; cells exposed to high phosphate levels produced red QDs. Fluorescence spectra of green and red biosynthesized NPs display broad emission spectra with peaks at 520 and 600 nm, respectively (Figure 1B).

Most biologically produced Cd-QDs described to date display broad emission peaks in the range of 410–650 nm after UV excitation (Espejo et al., 1988; Monras et al., 2012; Ulloa et al., 2016; Yan et al., 2017). Based on this, the effect of phosphate and cadmium concentrations on QDs biosynthesized was evaluated by determining the QDs-associated fluorescence produced by *A. thiooxidans* at the wavelength showing the emission peak for each condition used in the RSM experiments. Thus, response surfaces were obtained for different QDs synthesis conditions in supernatants at pH 3.5, varying the concentrations of cadmium and phosphate. The model obtained explains 72% of the variability of the response and the regression was significant (*p* < 0.05) (Figure 1C).

As shown in Figure 1C, a decreased production of fluorescent QDs is observed at low concentrations of phosphate, particularly at high cadmium concentrations. At low phosphate concentrations and high amounts of cadmium there is little emission and no evident formation of CdS QDs. However, when phosphate concentrations are maximal, an increase in fluorescence is observed even at high metal concentrations. Interestingly, the rise in QDs production is more pronounced at high concentrations of cadmium. The maximal fluorescence emission is obtained at high concentrations of phosphate and cadmium. Summarizing, results of Figure 1C indicate that phosphate favors the generation of fluorescent nanoparticles by *A. thiooxidans* at pH 3.5 (phosphate concentration had significant effect, *p* < 0.05%), which is in agreement with the results presented in Figures 1A,B.

To evaluate if the effect of phosphate and cadmium on the biosynthesis of QDs is related with differences on the structure or composition of QDs, the size and elemental composition of NPs purified from supernatants of cells exposed to 85 mM ${\text{PO}}_{4}^{3-}$/8.3 mM Cd^2+^ or 50 mM ${\text{PO}}_{4}^{3-}$/10 mM Cd^2+^ at pH 3.5 were determined (Figure S1). TEM images revealed that nanoparticle populations with an average size below 10 nm were observed in both synthesis conditions, a result that is in agreement with the NPs-size previously reported for CdS QDs biosynthesized by *A. thioxidans* at lower ${\text{PO}}_{4}^{3-}$ and Cd^2+^ concentrations (10 mM and 0.66 mM, respectively) (Ulloa et al., 2016). The presence of an organic matrix surrounding the NPs was observed in the TEM images. Accordingly, Dynamic Light Scattering determined hydrodynamic ratios below 50 nm in both samples, a result that is in agreement with the presence of an organic layer covering the QDs core (not shown). The EDS analysis revealed that biosynthesized QDs obtained from culture supernatants are mainly composed by Cd and S (Figure S1). As expected, the presence of P, C, and O was also observed in purified nanoparticles.

### Effect of phosphate on Cd uptake

**Figure 2:**
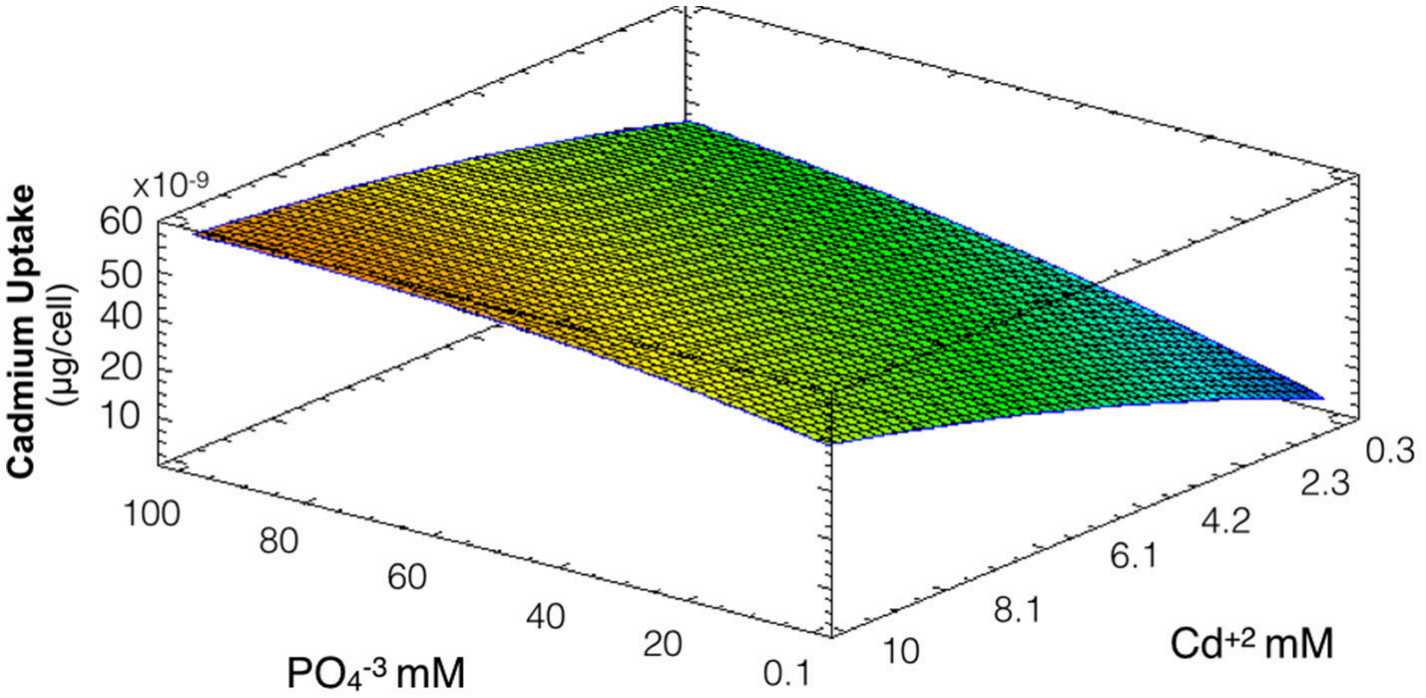
Response surface for a two variables model describing the effect of cadmium and phosphate culture concentrations on the uptake of Cd by *A. thiooxidans* cells at pH 3.5 (*R*^2^: 88%; *p* < 0.05). Higher concentrations of intracellular cadmium are represented in warmest colors, tending to red.

The same conditions used to evaluate biosynthesis of CdS-QDs were used to determine the influence of phosphate on cadmium uptake by *A. thiooxidans*. Bacterial pellets obtained in these conditions were washed several times to eliminate cell-adsorbed cadmium. In this case the model obtained explains 88% of the variability of the response and the regression was significant (*p* < 0.05). At low phosphate concentrations, bacterial cells show low capacity to incorporate cadmium (Figure 2). A direct relation between phosphate concentration and intracellular levels of cadmium was observed at all metal concentrations tested. Based on this, phosphate seems to be crucial modulating cadmium incorporation in *A. thiooxidans* (both factors had significant effect, *p* < 0.05).

The fact that intracellular Cd content increases at higher phosphate concentrations might be related to a co-transportation of cadmium and phosphate in *A. thiooxidans*. This phenomena of co-transportation of a metal with inorganic phosphate has been previously described in *Acinetobacter johnsonii* and acidophilic bacteria (Dameron et al., 1989; van Veen, 1997; Dopson et al., 2003; Alvarez and Jerez, 2004).

### Effect of phosphate on *A. thiooxidans* Cd^2+^ tolerance

**Figure 3:**
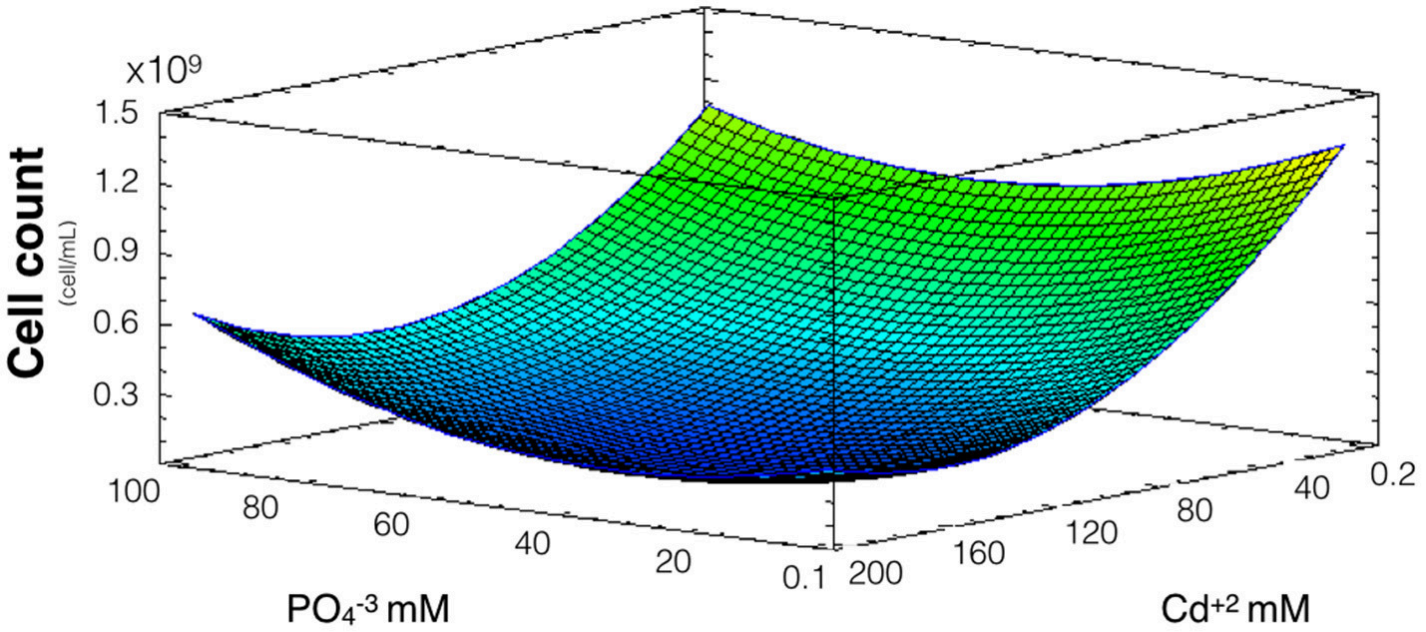
Response surface for a two variables model describing the effect of cadmium and phosphate culture concentrations on the number of *A. thiooxidans* cells. (*R*^2^: 68% *p* < 0.05).

Since phosphate has shown to influence QDs formation and cadmium uptake, and willing to address how phosphate influences CdS-QDs formation, the effect of phosphate on *A. thiooxidans* cadmium tolerance was evaluated. Cultures were incubated at pH 2.3 (optimal pH for metabolic activity) (Suzuki et al., 1999) with different concentrations of cadmium and phosphate for 8 days at 30°C, and the number of cells was determined. In this assay, the concentrations of cadmium used were higher than those needed for QDs biosynthesis (0.2–200 mM compared to 0.3–10 mM required for QDs formation). The model obtained explains 68% of the variability of the response and the regression was significant (*p* < 0.05) (Figure 3). As expected, a high number of cells were obtained at low levels of cadmium and phosphate does not generate a relevant effect on Cd-tolerance (only Cd concentration had significant effect, *p* < 0.05). However, when cadmium levels were higher, an increase in phosphate concentration generates a slight increase in the cell number, suggesting that phosphate protects bacteria from cadmium toxicity. The slight effect on cell number is probably related with phosphate-dependent metal complexation and/or with the biomineralization of CdS producing nano or micro particles (Ulloa et al., 2016).

Considering the results shown in Figures 2, 3, phosphate levels not only favor cellular uptake of the metal, but also increases cadmium tolerance. Altogether, these results suggest that CdS biosynthesis might be one of the strategies to immobilize cadmium and reduce their toxicity inside cells.

### Effect of phosphate and cadmium on polyP levels

**Figure 4:**
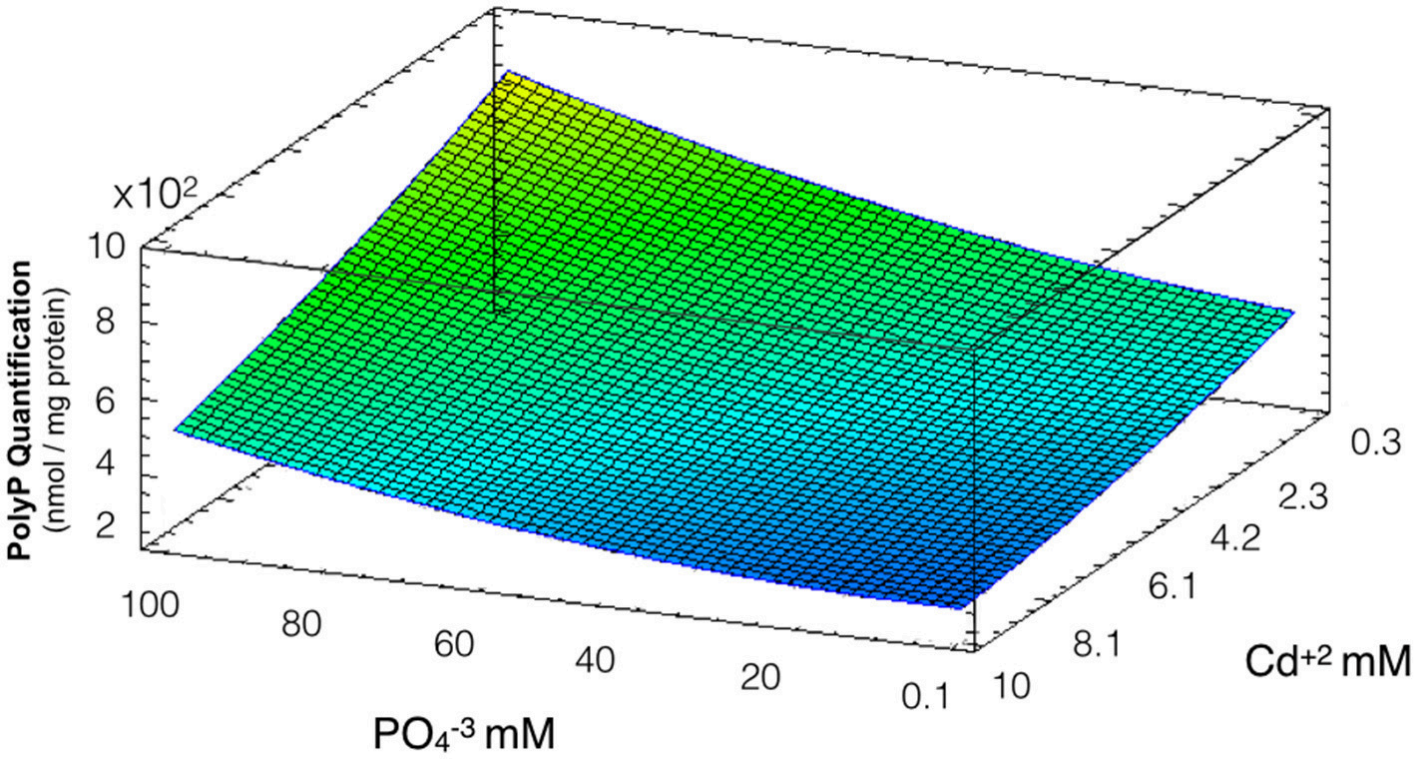
Response surface for a two variables model describing the effect of cadmium and phosphate on polyP levels during QDs formation conditions (*R*^2^: 86%, *p* < 0.05). All bacterial cultures display the same PolyP concentration in the absence of Cd and phosphate (they all become from the same stock), and the levels of PolyP were determined (nmol PolyP/mg protein) after 24 h exposure to different concentrations of cadmium and phosphate.

PolyP degradation is one of the main defense mechanism of *Acidithiobacillus* against the toxic effects of divalent metals like cadmium (Alvarez and Jerez, 2004; Ulloa et al., 2016). To evaluate the effect of phosphate on polyP levels and how this is related to the formation of QDs, a response surface measuring polyP levels of cells after exposure to the ${\text{PO}}_{4}^{3-}$ and Cd^2+^ concentrations used in biosynthesis conditions was constructed. The model obtained explains 86% of the variability of the response and the regression was significant (*p* < 0.05) (Figure 4). As expected, polyP concentrations are lower when Cd concentrations increase, a result that can reflect the degradation of the polymer in response to the metal stress in *A. thiooxidans* (${\text{PO}}_{4}^{3-}$ and Cd concentrations had significant effect, *p* < 0.05). Another results supporting the idea of a cellular response to Cd-stress are the increased cadmium uptake and the decreased cell viability observed at high cadmium concentrations in Figures 2, 3, respectively.

Low degradation of polyP was observed at high concentrations of phosphate and low levels of cadmium. Interestingly, the range of cadmium and phosphate concentrations favoring QDs biosynthesis determined in Figure 1 are those in which a high degradation of polyP was observed, suggesting a role of polyP in QDs formation.

## Discussion

The use of acidophilic extremophile bacteria to biosynthesize QDs is a topic of great industrial interest, not only because of their intrinsic resistance to both acidic pH and heavy metals (Cd^2+^), but also because of their potential applications in bioremediation of mining operations and the production of metal NPs with high commercial value.

In a recent work we have reported the use of acidophilic bacteria to biosynthesize CdS fluorescent nanoparticles, in a process that depends on the extracellular generation of sulfide in cultures supplemented with the biological thiols Cys or GSH (Ulloa et al., 2016). In general, abiotic oxidation of sulfide minerals (as pyrite or chalcopyrite) produces different inorganic sulfur compounds that are oxidized by acidophilic microorganisms to generate sulfuric acid (Rohwerder and Sand, 2007; Mangold et al., 2011; Yin et al., 2014). To date, most studies on sulfur metabolism in acidophilic bacteria have been related with sulfur oxidation, since this process is involved on their energetic metabolism. By comparative genomics it has been shown that bacteria of the *Acidithiobacillus* genus present different genes associated with sulfur oxidation (Valdés et al., 2008b; Mangold et al., 2011). In particular, the genomes of *A. thiooxidans* strains (19377 and A01) contains genes with similarity to sulfur oxidases of the SOX system (Valdés et al., 2008b; Zheng et al., 2015).

Regarding sulfur reduction, the existence of a functional sulfur assimilation pathway (SAP) in *Acidithiobacillus* has not been reported. However, the presence of genes involved in SAP was determined in *A. ferrooxidans* genome and their transcriptional response has been studied (Zheng et al., 2015). This process is particularly relevant for CdS QDs biosynthesis, since it is known that the generation of the reduced sulfur compound sulfide (S^2−^), is required for the interaction of sulfur with Cd^2+^ (Ulloa et al., 2016). An increase in the transcription levels of SAP genes in response to different Cd concentrations was determined recently in *A. ferrooxidans*, including genes coding for enzymes capable to produce S^2−^ such as sulfite reductase (CysJ, CysI) and O-acetylserine sulfhydrylase (CysM) (Zheng et al., 2015). Interestingly, Cys homologous genes are present in *A. thiooxidans* genome (not shown). These antecedents are in agreement with the increase in sulfide production observed in *Acidithiobacillus* cultures synthesizing CdS QDs previously reported by us (Ulloa et al., 2016).

Here, we report for the first time a detailed description of the CdS-QDs biosynthesis process by bacterial cells. In general, our results indicate that phosphate influences CdS-QDs biosynthesis in *A. thiooxidans* by favoring cadmium incorporation and tolerance. Interestingly, *A. thiooxidans* cells exposed to high phosphate concentrations preferentially produce red QDs (Figure 1A). This result is most probably related with the capacity of phosphate and phosphorylated molecules to improve cadmium-sulfide interaction and stabilize CdS nanostructures as has been previously shown in *E. coli* (Venegas et al., 2017). In addition, when different phosphate and cadmium concentrations were used to biosynthesize QDs at pH 3.5 (Figure 1C), no significant differences in size, structure and composition of QDs purified from cell supernatants were observed (Figure S1).

Also, obtained results indicate that polyP degradation favors QDs biosynthesis. This process is probably related with the generation of phosphates through polyP degradation. It has been reported that the PolyP-mediated production of intracellular phosphates is a key factor in heavy metal tolerance, particularly for Cu^2+^ and Cd^2+^ (Baillet et al., 1997; Van Dien and Keasling, 1998; Valdés et al., 2008b; Martínez-Bussenius et al., 2015). The presence of several genes potentially involved in cadmium resistance has been proposed in *Acidithiobacillus* based on sequence analyses of genomes (Valdés et al., 2008b), but to date direct evidences are scarce. Some potential efflux proteins with similarities to cadmium efflux channels (CadA) or ABC transporters were recently tested in *A. ferrooxidans* ATCC 23270 by means of transcriptional analysis (Chen et al., 2015; Zheng et al., 2015). An increase in expression of some of these genes was observed in presence of high Cd^2+^ concentrations. However, the function and participation of these genes on *A. ferrooxidans* Cd^2+^ resistance are still unclear. Also, putative genes that are part of the complexes of Resistance-nodulation-division (RND)-CzcCBA systems (*czcA*) were identified in two draft genome sequences: *A. thiooxidans* ATCC 19377 (GenBank accession number AFOH00000000) and ATCC A01 (GenBank accession AZMO00000000). These putative genes are annotated as NZ_AFOH01000140.1 and NZ_AZMO01000050.1, respectively. (RND)-CzcCBA systems are located in the cytoplasmic membrane and have shown to confer resistance to Cd^2+^, Zn^2+^, and Co^2+^ in Gram-negative bacteria (Dopson et al., 2003; Chen et al., 2015). These proteins may also play a role in the translocation of Cd^2+^ in the strain studied.

pH influenced the time required for *A. thiooxidans* QDs biosynthesis, and also the profile of fluorescence of the nanoparticles. Since *Acidithiobacillus* is metabolically more active at pH 3.5 than 7.0, the improved QDs synthesis at acidic pH could be associated with increased ATP requirements for processes like phosphate uptake and metal tolerance. This would explain why the biosynthesis of CdS-QDs requires 24 h at pH 3.5 and 48 h at pH 7.0. This effect could also be related to the protonation state of phosphate hydroxyl groups at the different pHs, influencing the flux of phosphate through the membrane. There is a phosphate-specific transport system described in *A. ferrooxidans* (Pst) (Vera et al., 2003; Valdés et al., 2008a; Orell et al., 2012), also present in *E. coli* (Aguena and Spira, 2009), that might be responsible for at least part of the Pi uptake. Genomic analysis revealed that this system is present in *A. thiooxidans* (Martínez-Bussenius et al., 2017). There is also a putative Pho84-like protein in *A. ferrooxidans* with 26% identity with *S. cerevisiae* Pho84 (Alvarez and Jerez, 2004). Pho84 is a P_i_:H^+^ symporter that has a maximum activity at acidic extracellular pH (Zvyagilskaya and Persson, 2003). It is possible that *A. thiooxidans* ATCC 19703 also expresses a Pho transporter responding to the pH conditions of the media. In support of this, the existence of a putative *pho84* gene was recently described in *A. thiooxidans* by genomic analysis (Martínez-Bussenius et al., 2017).

We also observed that phosphate levels modulate the intracellular cadmium-content in *A. thiooxidans*, a phenomenon that was also related with the capacity to biosynthesize CdS-QDs. A high rate of cadmium incorporation was determined at pH 3.5 in *A. thiooxidans*, a result that is in agreement with antecedents indicating that cadmium uptake is favored in bacterial cells grown at optimal pH (Baillet et al., 1997).

Fluorescent supernatants reflect the extracellular production of Cd-QDs, as has been previously described in other microorganisms (Jacob et al., 2016). In this particular case, extracellular QDs produced by *A. thiooxidans* must tolerate the acidic conditions required for biosynthesis, a very unusual characteristic of QDs that was previously reported by our group in QDs biosynthesized by bacteria of the genus *Acidithiobacillus* (Ulloa et al., 2016).

**Figure 5:**
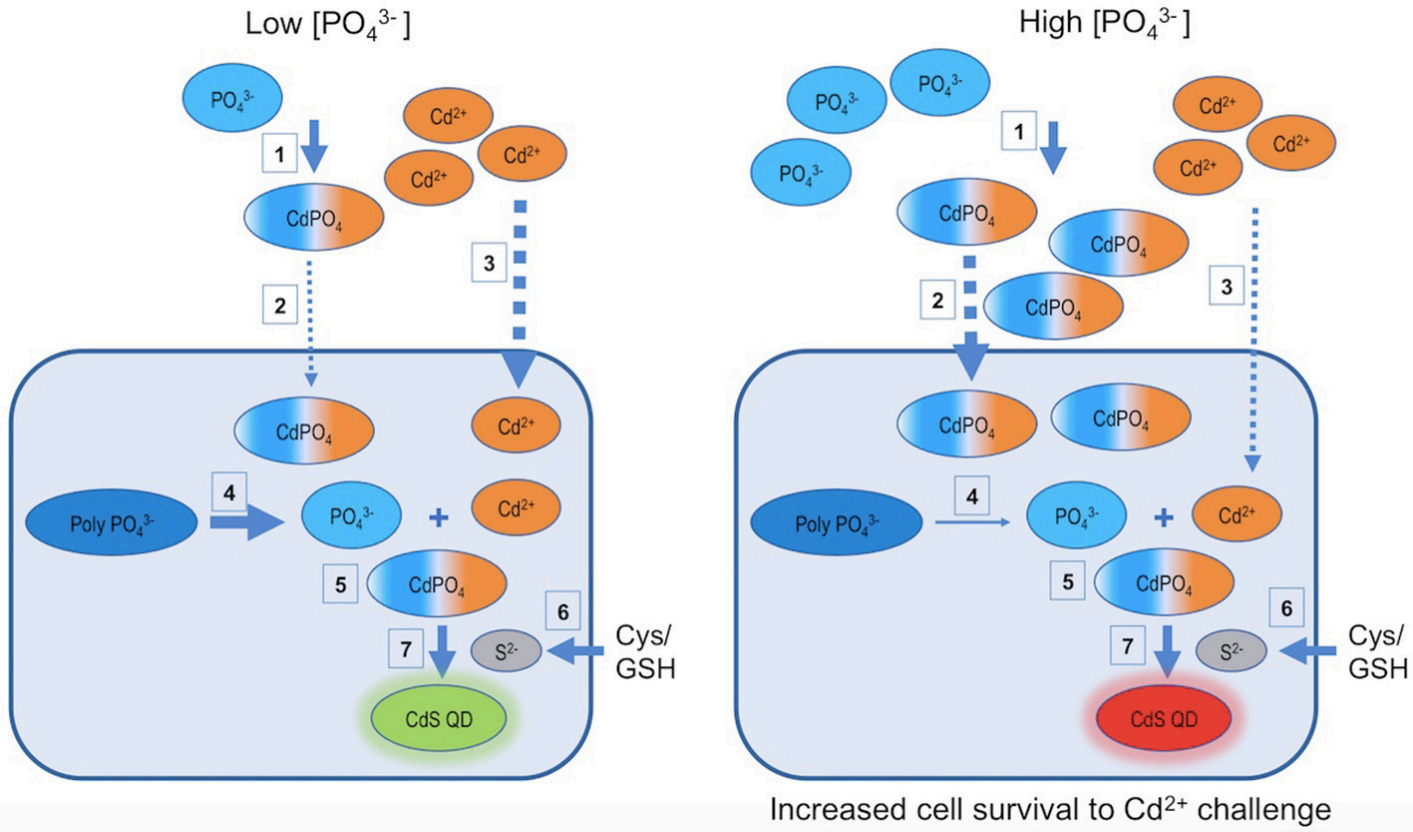
Models for phosphate dependent CdS-QDs biosynthesis by *A. thiooxidans* ATCC 19703. Based on the results of the present work, two models describing the phosphate dependent CdS QDs biosynthesis process at low and high ${\text{PO}}_{4}^{3-}$ concentrations are proposed. GSH, glutathione; Cys, cysteine; ${\text{PolyPO}}_{4}^{3-}$, polyphosphates; CdPO_4_, cadmium phosphate complex.

So, based on obtained results we propose a model explaining the effect of low and high phosphate concentrations on the biosynthesis of Cd-based QDs in *A. thiooxidans* ATCC 19703 (Figure 5). As described in Figure 1, the biosynthesis of QDs is favored when phosphate is incorporated to the culture. (1) Phosphate can interact with Cd^2+^ forming metal complexes inside and outside cells (Dopson et al., 2003; Mangold et al., 2011). (2) The generation of cadmium-phosphate complexes favors the accumulation of cadmium inside cells (results of Figure 2). (3) This accumulation could be consequence of an increase in Cd-uptake (as cadmium-phosphate complexes and also as Cd^2+^), a diminished Cd-secretion and/or the production of QDs. In addition, this interaction decreases the toxicity of Cd^2+^ ions (results of Figure 3), most probably by avoiding the interaction with cellular targets such as proteins and cellular thiols (Holmes et al., 1997; Ulloa et al., 2016). (4) Intracellular polyP participates in the biosynthesis of QDs acting as an additional source of ${\text{PO}}_{4}^{3-}$ required for biosynthesis, but also by protecting the cell from Cd^2+^ toxicity. (5) In addition to these effects, there is evidence suggesting that intracellular QDs co-localize with polyP inside cells (Monras et al., 2012), probably as consequence of a direct interaction between them, or via the generation of Cd-phosphate complexes that can act as nucleation sites for nanocrystal growth. (6) Regarding the production of sulfur required to synthesize CdS, several sources of thiols, like glutathione or cysteine, can act as precursors in different microorganisms including *A. thiooxidans* (Valdés et al., 2008b; Bao et al., 2010; Ulloa et al., 2016). This process has been mostly associated to the activity of enzymes involved in S metabolic pathways such as cysteine desulfhydrases (Valdés et al., 2008b; Narayanan and Sakthivel, 2010; Ulloa et al., 2016). (7) Sulfur can interact with Cd^2+^ or cadmium-phosphate complexes inside and outside cells, generating nanocrystals (Gallardo et al., 2014; Plaza et al., 2016; Ulloa et al., 2016). Biosynthesized QDs are probably stabilized by cellular biomolecules like proteins and/or biological thiols such as GSH/cysteine (Mi et al., 2011; Ulloa et al., 2016; Yan et al., 2017).

Finally, a potential role of CdS QDs biosynthesis in bacterial resistance to Cd^2+^ can be proposed. More experiments must be done to validate this idea, but the results of this study allow us to propose that the generation of these nanostructures (and also probably of microparticles) is another alternative of cells to sequester this toxic metal and avoid its interaction with biomolecules.

## Author contributions

GU, EF, and JP-D: conceived and designed the study; GU, MC, NB, RE-G, and MA: performed the experiments; CQ, BE, RE-G, SÁ, DB, and JP-D: analyzed the data and prepared the manuscript.

### Conflict of interest statement

The authors declare that the research was conducted in the absence of any commercial or financial relationships that could be construed as a potential conflict of interest.

## Supplementary material

Figure S1 HR-TEM and EDS analysis of CdS-QDs biosynthesized by *A. thiooxidans* ATCC 19703 at pH 3.5 in presence of two ${\text{PO}}_{4}^{3-}$ and Cd^2+^ concentrations used to construct the surface response of Figure 1C. QDs biosynthesized by *A. thiooxidans* ATCC 19703 after exposure to 85 mM ${\text{PO}}_{4}^{3-}$/8.3 mM Cd^2+^ **(A,B)** and 50 mM ${\text{PO}}_{4}^{3-}$/10 mM Cd^2+^ **(C,D)** were purified from culture supernatants and characterized by HR-TEM and EDS.

Table S1 RSM experimental conditions. Two experimental conditions were used for RSM construction; MIC conditions and Biosynthesis conditions. The nine coded conditions used and the corresponding values of variables for the two experimental conditions are indicated. The biosynthesis of NPs was evaluated in supernatants of cell cultures exposed 24 h to the indicated Cd^2+^ and ${\text{PO}}_{4}^{3-}$ concentrations at pH 3.5. Rows 4 and 9 (highlighted) correspond to those experimental conditions in which size and composition of biosynthesized QDs were characterized by TEM and EDS, respectively (Figure S1).
