# Supplementary figures and images for: Phosphate Favors the Biosynthesis of CdS Quantum Dots in Acidithiobacillus thiooxidans ATCC 19703 by Improving Metal Uptake and Tolerance
Source: Front Microbiol. 2018 Feb 20;9:234. doi: 10.3389/fmicb.2018.00234 (PMC5826283; doi:10.3389/fmicb.2018.00234)

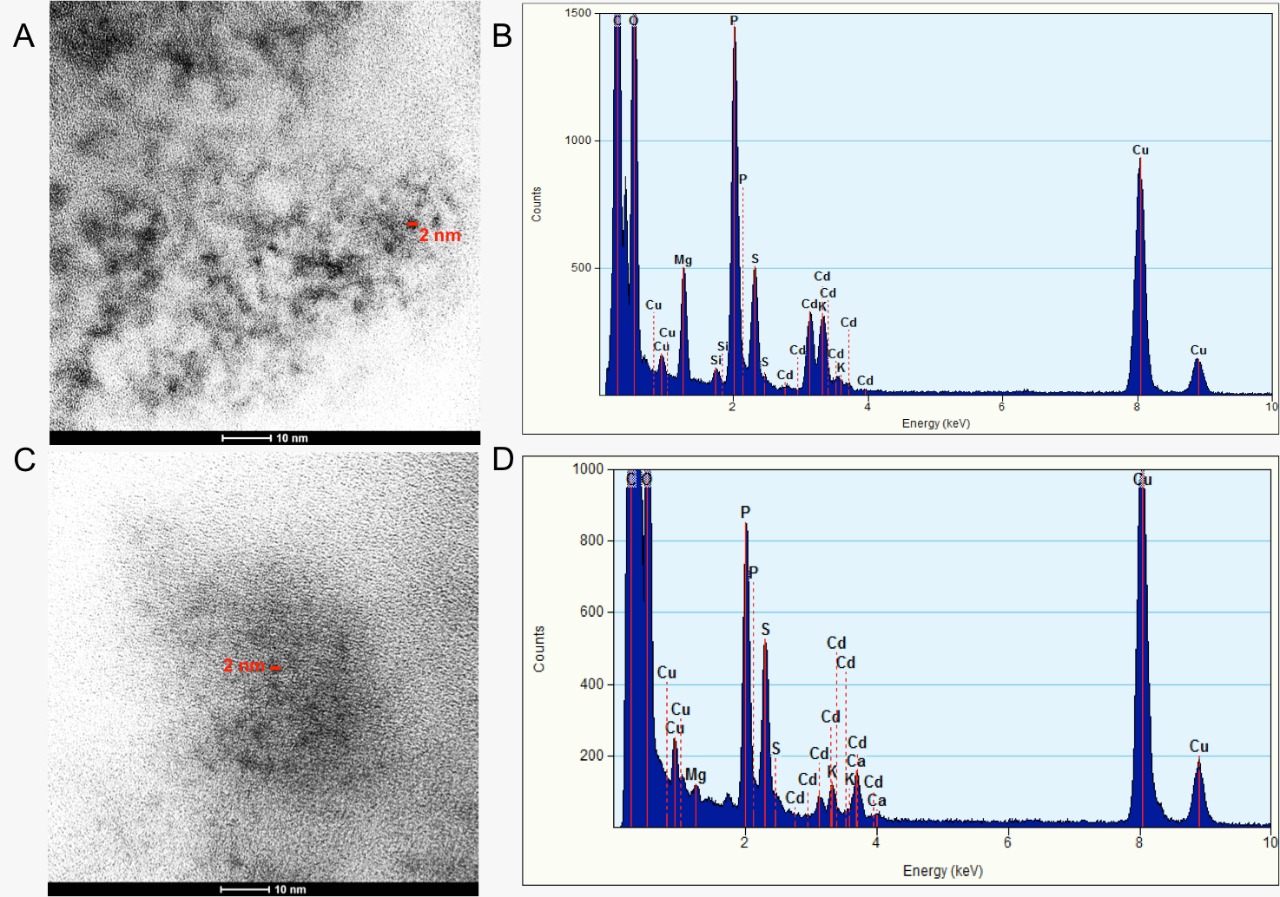

Supplement: Figure S1 — HR-TEM and EDS analysis of CdS-QDs biosynthesized by A. thiooxidans ATCC 19703 at pH 3.5 in presence of two PO43- and Cd2+ concentrations used to construct the surface response of Figure 1C. QDs biosynthesized by A. thiooxidans ATCC 19703 after exposure to 85 mM PO43-/8.3 mM Cd2+ (A,B) and 50 mM PO43-/10 mM Cd2+ (C,D) were purified from culture supernatants and characterized by HR-TEM and EDS. [file Image1.JPEG]
