# Supplementary material for: Phosphate Favors the Biosynthesis of CdS Quantum Dots in Acidithiobacillus thiooxidans ATCC 19703 by Improving Metal Uptake and Tolerance
Source: Front Microbiol. 2018 Feb 20;9:234. doi: 10.3389/fmicb.2018.00234 (PMC5826283; doi:10.3389/fmicb.2018.00234)
Supplement: Table S1 — RSM experimental conditions. Two experimental conditions were used for RSM construction; MIC conditions and Biosynthesis conditions. The nine coded conditions used and the corresponding values of variables for the two experimental conditions are indicated. The biosynthesis of NPs was evaluated in supernatants of cell cultures exposed 24 h to the indicated Cd2+ and PO43- concentrations at pH 3.5. Rows 4 and 9 (highlighted) correspond to those experimental conditions in which size and composition of biosynthesized QDs were characterized by TEM and EDS, respectively (Figure S1). [file Table1.docx]

| **Experimental conditions (mM)** | | | | | | | **Results of RSM in experimental conditions** | | | | | | | |
| --- | --- | --- | --- | --- | --- | --- | --- | --- | --- | --- | --- | --- | --- | --- |
| **Coded levels** | | | **MIC conditions** | | **Biosynthesis conditions** | | **MIC conditions** | | **Biosynthesis conditions** | | | | | |
| **Run** | **x1** | **x2** | **PO_4_^3-^** | **Cd^2+^** | **PO_4_^3-^** | **Cd^2+^** | **Cell counts** | **SD** | **Fluorescence** | **SD** | **Cd uptake** | **SD** | **PolyP quantification** | **SD** |
|  |  |  |  |  |  |  | **(cell/mL)** |  | **(f.u.)** |  | **(μg/cell)** |  | **(nmol/mg protein)** |  |
| 1 | -1 | -1 | 15 | 29 | 15 | 1,4 | 1,30E+09 | 4,80E+03 | 41770 | 3,20E+03 | 1,10E-08 | 1,47E-09 | 405,8 | 20,6 |
| 2 | 1 | -1 | 85 | 29 | 85 | 1,4 | 1,50E+09 | 4,70E+05 | 71200 | 5,90E+03 | 1,80E-08 | 5,86E-10 | 681,2 | 24,8 |
| 3 | -1 | 1 | 15 | 171 | 15 | 8,3 | 1,80E+08 | 6,40E+06 | 22990 | 1,50E+03 | 1,90E-08 | 4,82E-09 | 135 | 7,2 |
| **4** | **1** | **1** | **85** | **171** | **85** | **8,3** | **1,00E+09** | **1,40E+07** | **73400** | **2,70E+03** | **2,10E-08** | **1,73E-09** | **248,3** | **9,5** |
| 5 | -1.41 | 0 | 0,1 | 100 | 0,1 | 4,8 | 8,30E+08 | 4,20E+06 | 31009 | 1,10E+03 | 1,40E-08 | 5,61E-09 | 33 | 5,1 |
| 6 | 1.41 | 0 | 100 | 100 | 100 | 4,8 | 2,00E+08 | 2,80E+06 | 32508 | 1,50E+03 | 2,00E-08 | 4,93E-09 | 631,1 | 27,4 |
| 7 | 0 | -1.41 | 50 | 0,2 | 50 | 0,33 | 1,30E+09 | 1,50E+07 | 35006 | 2,00E+03 | 1,30E-08 | 3,79E-09 | 389,4 | 21,5 |
| 8 | 0 | 1.41 | 50 | 200 | 50 | 10 | 4,00E+08 | 3,50E+06 | 35677 | 1,20E+03 | 2,20E-08 | 5,97E-09 | 152,3 | 4,7 |
| **9** | **0** | **0** | **50** | **100** | **50** | **4,8** | **1,50E+08** | **3,50E+03** | **28908** | **1,10E+03** | **1,90E-08** | **1,53E-09** | **253,5** | **14,1** |
